# Pyrrolizidine Alkaloids as Hazardous Toxins in Natural Products: Current Analytical Methods and Latest Legal Regulations

**DOI:** 10.3390/molecules29143269

**Published:** 2024-07-10

**Authors:** Agnieszka Lis-Cieplak, Katarzyna Trześniowska, Krzysztof Stolarczyk, Elżbieta U. Stolarczyk

**Affiliations:** 1 Spectrometric Methods Department, National Medicines Institute, Chełmska 30/34, 00-725 Warsaw, Poland; a.lis@nil.gov.pl (A.L.-C.); k.trzesniowska@nil.gov.pl (K.T.); 2 Faculty of Chemistry, University of Warsaw, Pasteura 1, 02-093 Warsaw, Poland; kstolar@chem.uw.edu.pl

**Keywords:** pyrrolizidine alkaloids, chromatographic methods, electrochemical sensors, herbal products, carcinogenesis, genotoxicity, public health

## Abstract

Pyrrolizidine alkaloids (PAs) are toxic compounds that occur naturally in certain plants, however, there are many secondary pathways causing PA contamination of other plants, including medicinal herbs and plant-based food products, which pose a risk of human intoxication. It is proven that chronic exposure to PAs causes serious adverse health consequences resulting from their cytotoxicity and genotoxicity. This review briefly presents PA occurrence, structures, chemistry, and toxicity, as well as a set of analytical methods. Recently developed sensitive electrochemical and chromatographic methods for the determination of PAs in honey, teas, herbs, and spices were summarized. The main strategies for improving the analytical efficiency of PA determination are related to the use of mass spectrometric (MS) detection; therefore, this review focuses on advances in MS-based methods. Raising awareness of the potential health risks associated with the presence of PAs in food and herbal medicines requires ongoing research in this area, including the development of sensitive methods for PA determination and rigorous legal regulations of PA intake from herbal products. The maximum levels of PAs in certain products are regulated by the European Commission; however, the precise knowledge about which products contain trace but significant amounts of these alkaloids is still insufficient.

## 1. Introduction

Natural products have been used for centuries in traditional medicine to treat and prevent diseases. Nowadays, many drugs are obtained from plant raw material, especially from medicinal herbs, and natural substances isolated from medicinal plants are considered candidates for new drugs [1]. Moreover, other natural sources, including fungi, lichens, bacteria, and marine organisms, also provide valuable material for the pharmaceutical industry. On the other hand, due to the growing interest in healthy lifestyles, the use of supplements and medicinal products of plant origin is becoming popular among consumers. The market for foods classified as dietary supplements is developing dynamically. There is a misconception that these products are always safe and side-effect free. Importantly, these products are consumed without proper medical supervision. To make dietary supplements safe for consumers, relevant legal regulations and market control are needed.

Contamination of food products and plant-based medicines with pyrrolizidine alkaloids (PAs) emerged recently as a significant issue at the international level [2,3,4,5,6,7,8,9,10,11,12,13,14,15,16,17,18] as PAs have been detected in honey and plant material such as tea, herbs, vegetables, cereals, salads, and spices. Therefore, it becomes the subject of numerous discussions, research projects, reports, and legal regulations. Numerous aspects of PA chemistry, metabolism, and toxicity were discussed in high-quality review articles [8,15,19,20,21,22,23,24,25,26,27,28,29,30]. The occurrence of PAs in food and herbal products is a very important problem, and due to the health hazards, effective methods of their identification and determination should be constantly developed. Techniques such as high-performance liquid chromatography (HPLC), gas chromatography (GC), and thin-layer chromatography (TLC) are used for the detection and identification of PAs [31]. Spectroscopic techniques are utilized as well [31]. However, these methods do not provide sufficient identification accuracy or sensitivity to determine PA contamination on trace levels. For that purpose, more advanced methods were recently developed. These protocols mostly rely on the application of liquid chromatography combined with mass spectrometry detection (LC-MS) [31,32]. LC-MS methods combine reliability and high sensitivity with ease of sample preparation. This is because LC-MS ensures very selective PA identification and determination, even in complicated matrixes. Therefore, recently, it has become the method of choice in PA analysis [33,34,35,36,37,38,39,40,41].

This review provides information on the occurrence of PAs in the environment along with insight into secondary pathways of PA contamination of plant products. It focuses on analytical techniques applied for PA determination in medicinal and food products of plant origin. Special emphasis is put on liquid chromatography (LC) methods combined with mass spectrometry (MS) detection. However, other methods are discussed as well. Recently published reviews [25,27,28,29,41,42,43] summarize methods for determining PA, mainly methods with MS detection using various types of analyzers, which are characterized by different levels of sensitivity. The most modern and sensitive type of detector is currently Orbitrap, and there are relatively few reports on methods using this type of analyzer in other reviews. This review presents PA testing methods using both basic types of analyzers and the most modern ones, based on the latest literature reports. This review also provides much more detailed information on the mechanisms of PA-induced cytotoxicity and genotoxicity, as well as their LD_50_ values, which are not included in existing reviews. It also provides a broad summary of European regulations regarding these dangerous pollutants.

## 2. Natural Occurrence of PAs, Health Risks, and Possible Routes of Human Intoxication

In general, alkaloids are a diverse class of naturally occurring organic compounds. They are characterized primarily by the presence of at least one nitrogen atom in the structure. The inherence of nitrogen in the form of a heteroatom in the rings, in the form of an amino group or, less often, an amide group, causes the basicity of these compounds. This broad group also includes related ones that have neutral or even slightly acidic properties. Alkaloids may also contain other heteroatoms such as sulfur and, less commonly, phosphorus, chlorine, and bromine [44]. These compounds are synthesized by a wide range of organisms; they can be found in bacteria, fungi, plants, and animals [45,46]. Alkaloids have a wide range of pharmacological activities, such as antimalarial, antiasthmatic, anticancer, cholinomimetic, vasodilatory, antiarrhythmic, analgesic, antibacterial, and antihyperglycemic effects. Due to their strong biological activity, many alkaloids are used in traditional or modern medicine or serve as starting points for drug discovery [47].

**Figure 1:**
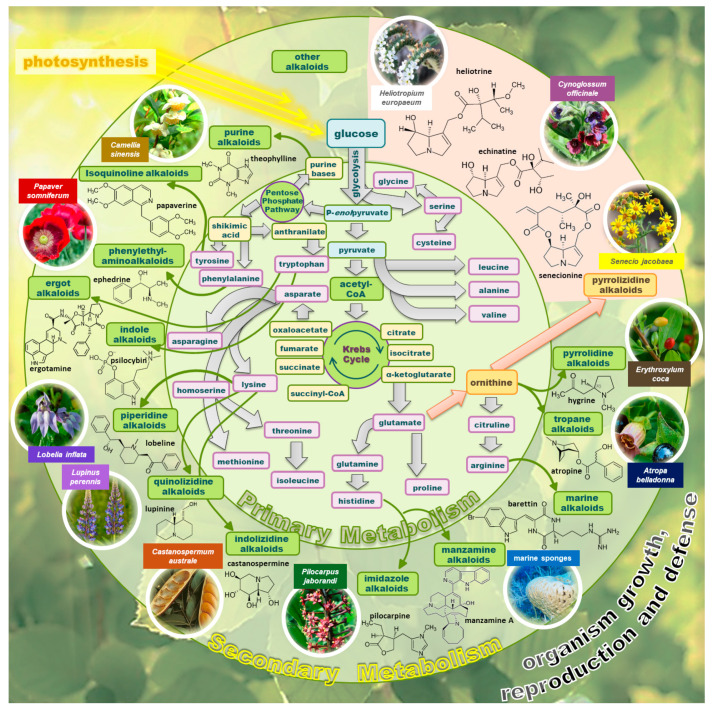
A block scheme of the alkaloid biosynthesis as a secondary metabolism of plants. The pathway of PAs biosynthesis is marked with orange arrows and boxes. Based on [21]. The figure was prepared using GIMP 2.8.14 (GNU General Public License) software.

Alkaloid synthesis is a secondary metabolic process. The necessary compounds for alkaloid synthesis are pyruvic acid and acetyl-CoA. Figure 1 shows the synthesis of alkaloids in the metabolic system of plants. The synthesis of alkaloids in the plant is a multi-step process. The first stage is photosynthesis, which produces the monosaccharide D-glucose, which is used as a substrate in the next stage of the Krebs cycle. PAs are compounds containing nitrogen in fused heterocyclic rings, produced in the ornithine metabolism pathway (see Figure 1), while L-ornithine is derived from L-glutamate [21]. There were hundreds of structurally different PAs discovered. The occurrence of PAs, their propagation, toxicity, and chemistry are described in detail in the next paragraphs.

PAs are synthesized by a wide variety of plant species. They were identified in over 6000 plants [48], in families: *Boraginaceae* (all genera), *Asteraceae* (*Senecioneae*, *Eupatorieae*), and *Fabaceae* (*Crotalaria*), as natural toxins providing protection against animals feeding on plants [23,49]. PA-containing plants are common weeds and are considered invasive and harmful to the environment because they may contaminate the raw plant material. On the crop fields, PA-containing plants and their parts or seeds can contaminate soil and get into harvested cereals, herbs, or vegetables. Accidental mixing of PA-containing plants with plants intended for fodder may lead to contamination of prepared feeds and grains that are subsequently eaten by animals. That makes food of animal origin, such as milk and eggs, a health hazard [23,48,49,50,51]. Bees can ingest PAs containing pollen, and then they produce contaminated honey [52]. Therefore, it leads to PA contamination of the whole food chain.

**Figure 2:**
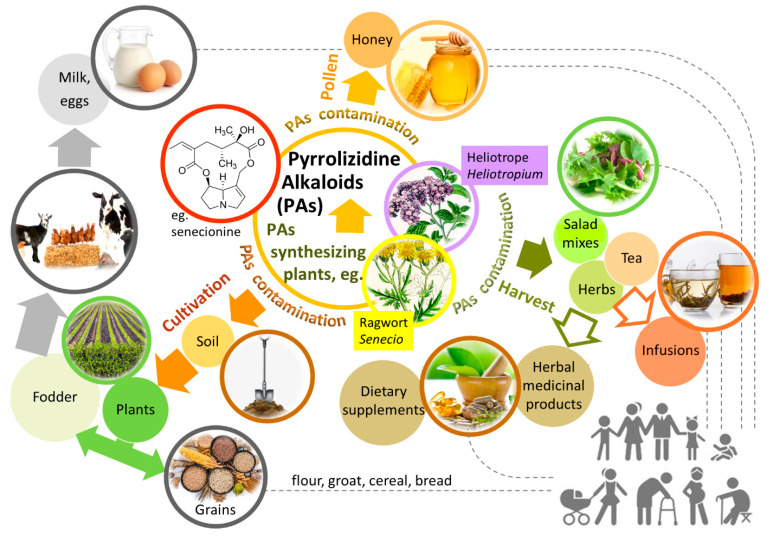
The possible pathways of human exposure to PAs. The figure was prepared using GIMP 2.8.14 (GNU General Public License) software.

The European Food Safety Authority (EFSA) recognized PAs as potential toxic components of feed and food, which can become a significant public health problem due to the high risk of contamination of food of plant or animal origin [9]. Possible routes of intoxication with PAs are shown in Figure 2. Generally, PA intoxication is possible through ingestion of PA-containing herbal products or PA-contaminated foods, such as tea, herbs, vegetables, spices, and salads. PA-containing plants are numerous and widespread. Human intoxication can occur through the consumption of contaminated basic food products and some herbal remedies [15].

## 3. Chemistry of PAs

**Figure 3:**
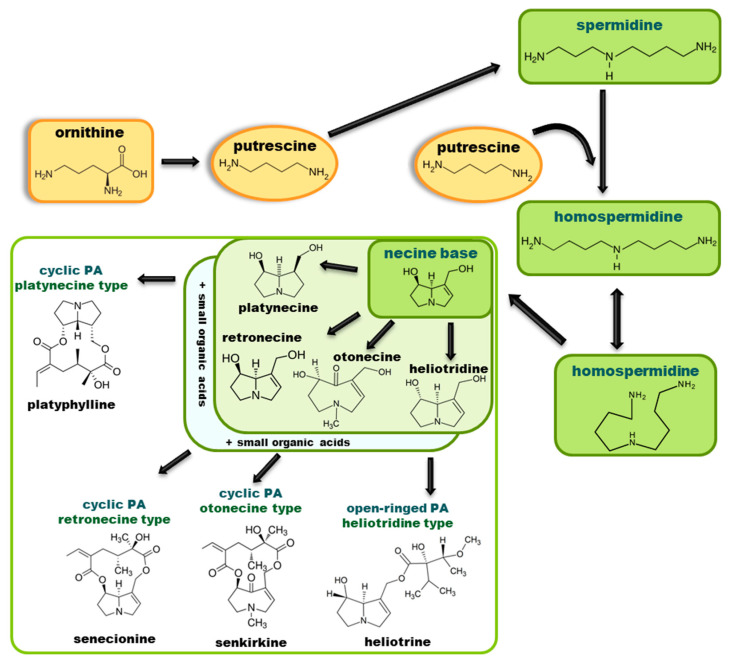
Biosynthesis of the most representative examples of PAs. Based on [19,55]. The figure was prepared using GIMP 2.8.14 (GNU General Public License) software.

So far, more than 500 PAs have been found and their structures determined [53]. Taking into account the form of *N*-oxides, over 900 structures are known. PAs are heterocyclic compounds, they include a group of basic ester compounds (mono- and diesters), which structurally include a combination of amino alcohols with mono- or dicarboxylic acids [22]. They are pyrrolizidine or necine derivatives, esters, and diesters [21]. PAs undergo acidic or basic hydrolysis, giving basic necine-type amino alcohols that can be assigned as PA groups according to the necine base: otonecine, retronecine, heliotridine, and platynecine. The biosynthesis of PAs begins with the amino acid ornithine, which leads to the generation of putrescine and then spermidine. One molecule of putrescine and spermidine is transformed into homospermidine [54]. Homospermidine is deaminated and creates the necine base skeleton, esterified with a necic acid [55]. The necine base forms esters with small organic acids and generates cyclic PAs (retronecine and otonecine type) and open-ringed PAs (heliotridine type) (Figure 3). In detail, a molecule of PAs consists of the core structure—pyrrolizidine, a bicyclic aliphatic hydrocarbon consisting of two fused five-membered rings with a nitrogen atom between them and in many structures with a double bond in the 1,2 positions (sub-group of PAs, 1,2-unsaturated). The main toxic effects of PAs are on the liver and lungs. 1,2-unsaturated PAs are genotoxic and cause liver cancer in experimental animals. The Scientific Panel on Contaminants in the Food Chain (CONTAM Panel) of the European Food Safety Authority published a scientific opinion on the risks to public health related to the presence of pyrrolizidine alkaloids in food and feed. The CONTAM Panel concluded that only 1,2-unsaturated pyrrolizidine alkaloids are toxic and may act as genotoxic carcinogens in humans [9,56]. The necine base is often retronecine, heliotridine, or otonecine. Necic acids are varied organic acids, when they are dicarboxylic, they form macrocyclic PAs. PAs demonstrate great structural diversity. With the large number of necic acids, which can be combined with a set of necine bases, a huge structural diversity of PAs is possible [22]. Moreover, modifications including *N*-oxidation of the tertiary nitrogen of the necine base, hydroxylation of the necine base and the necic acid, and acetylation of hydroxy groups further enhance these possibilities. Chemical structures of PAs listed in Annex 1 to the Commission Regulation (EU) 2023/915 [57] are presented in Table 1. The chemical structures of PAs, which should also be monitored in food and feed, according to the EFSA opinion [8,9], are presented in Table 2. *N*-oxidation is a special type of modification because it is reversible. *N*-oxidation of the tertiary amine nitrogen significantly changes the properties of a native PA. The main point is that PANOs become more polar and highly water soluble. In plants, what is most important is that the major fraction of PAs is present as PANOs [22].

The determination of PANO concentration is necessary because, in vivo, these *N*-oxides can be biotransformed into the corresponding PA-free bases. This process takes place after ingestion in the gastrointestinal tract and in the liver, mediated by, respectively, the intestinal microbiota and hepatic cytochrome P450 monooxygenases [58]. When reduced, *N*-oxide-derived PAs are subsequently metabolized to the hepatotoxic pyrrole. Therefore, the total PA content of the tested material may be underestimated unless both PA and PANO contents are determined.

## 4. Toxicity of PAs and Regulation Limits

**Figure 4:**
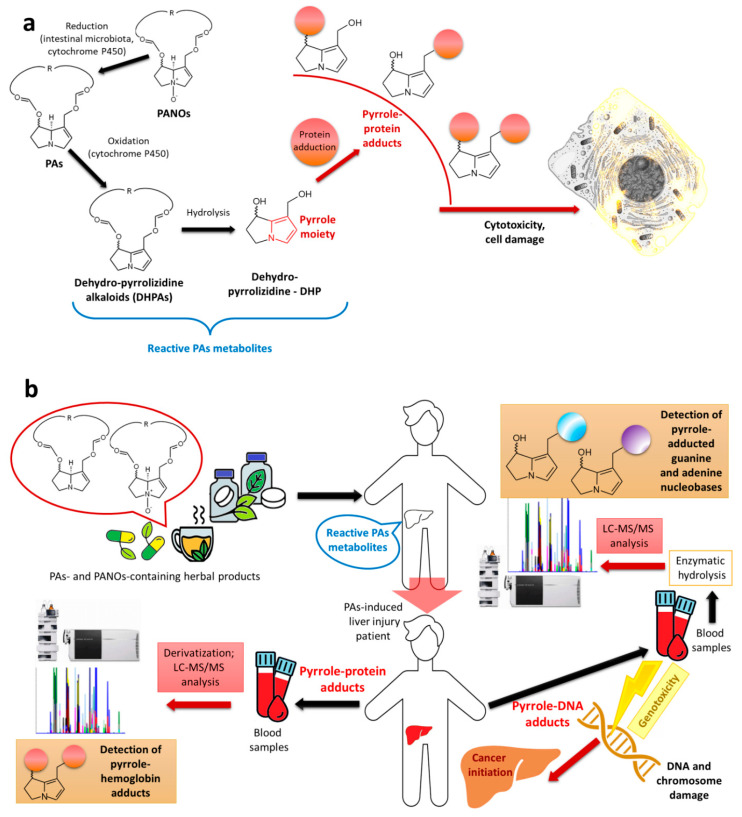
Mechanism of metabolic processes of PAs, which leads to hepato- and cytotoxicity (**a**) and hepato- and genotoxicity (**b**). Adapted from [61,62]. The figure was prepared using GIMP 2.8.14 (GNU General Public License) software.

PAs are potentially hepatotoxic compounds that lead to human poisoning through the food chain (such as tea, herbs, botanical preparations, spices, and vegetables). The toxicity of PAs depends on their physical properties and their metabolism in the liver [59]. PAs very often produce pyrrolizidine alkaloid *N*-oxides (PANOs), of lower toxicity, that cannot be directly converted to hydroxypyrrolidines. PANOs generally exhibit low toxicity but undergo toxic processes in vivo and cause toxification through biotransformation to the corresponding PAs [60]. PANOs are reduced to free bases in the intestines after their ingestion, which was described further. The hepato- and cytotoxicity mechanisms of PAs are shown in Figure 4.

The oxidation of PAs is a metabolic process that occurs in the liver. This process is crucial as it leads to the formation of reactive PA metabolites [62] (see Figure 4a). During oxidation processes in the liver, a hydroxyl group is attached to the alkaloid molecule and the carbon adjacent to the nitrogen atom. The alkaloid thus formed is unstable and is immediately dehydrated into the dehydro-pyrrolizidine alkaloids (DHPAs). As a result, a second double bond is formed in the necine molecule. At a later stage, after hydrolysis, an aromatic pyrrole moiety is generated (dehydropyrrolizidine—DHP), with the ester groups removed. In the final process, there are formed pyrrole–protein adducts and pyrrole-DNA adducts, which are responsible for cytotoxicity and genotoxicity (see Figure 4b) [61,63]. PA-induced liver injury is suspected to be associated with the consumption of PA-containing herbal products, and the pyrrole–DNA adducts were detectable in patients’ blood samples [61].

PAs may cause acute toxicity, mutagenicity, chromosomal aberrations, the formation of abnormal cross-links between DNA strands and DNA–protein bonds, and megalocytosis [23,64,65]. They are responsible for: the formation of cancer cells [56,66,67], disturbances of the liver metabolism [23,66], liver necrosis [19], fibrosis [19], cirrhosis [19], photosensitization [19,23], diarrhea [19,23], incoordination [8,28], aggressive behavior [8], body weight loss [8,23], loss of appetite [8,23]. Poisoning with PAs is usually asymptomatic. By the time symptoms of liver damage appear, the disease process has already developed and caused death in a short time [23,68]. The LD_50_ values of the most important PAs are known [2]. However, the increase or inhibition of cytochrome P450 activity by drugs may also change the toxicity of PAs. PA metabolites react with SH groups located in glutathione or cysteine. Therefore, a diet rich in glutathione, taurine, cysteine, and methionine may reduce the toxicity of ingested PAs [69]. PA toxicity also depends on exposure time, dose, and the organism’s susceptibility. PAs cause acute toxicity within 1–6 days, while doses of 0.1 mg/kg body weight per day cause chronic toxicity. In humans, the toxic dose ranges from 0.1–10 mg/kg body weight per day [10]. Table 3 shows the LD_50_ of the more important PAs studied in vivo in animal models [2] and predicted using the computer software TOPKAT (Discovery Studio 2019 (Accelrys, Inc., San Diego, CA, USA). [70].

On 31 May 2016, the regulatory authorities of the EU (European Medicines Agency—EMA and Herbal Medicinal Products Committee—HMPC) issued a public statement regarding the contamination of herbal medicinal products/traditional products by PAs [10]. Following a review of the available data, the EMA (HMPC) considered a harmonized approach to implementing appropriate controls for the markets in EU countries. A contamination level of herbal medicinal products leading to a daily intake of a maximum of 1.0 μg PAs per day during a transitional period of 3 years was considered acceptable from a public health point of view. After this period, producers of herbal medicinal products should be required to take the necessary measures to reduce the contamination to a level resulting in a daily intake not exceeding 0.35 μg PAs per day [10,14,71]. The report concluded that contamination of herbal products (food or medicines) with PAs is not a new matter, but new, sensitive analytical methods can now detect very low levels of PAs. Tea and herbal teas are the largest contributors to human exposure to pyrrolizidine alkaloids, as well as pollen-based supplements. It was found that the exposure to pyrrolizidine alkaloids associated with the consumption of honey is lower. It has also been found that herbal dietary supplements may contribute significantly to human exposure to PAs, but incidence data are insufficient [10]. Generally, the human intake of PAs through food and herbal medicinal products has probably remained constant over the last few years, and statistically, the incidence of liver hemangiosarcoma in humans is very low. EMA has emphasized that once the problem with PA contamination of herbal medicinal products has been identified, regulatory actions to mitigate the problem must be considered.

The European Food Safety Authority (EFSA) prepared in 2016 a report on chronic and acute dietary exposure to PAs in the European population through the consumption of foods of plant origin [11]. This scientific report focuses on the 28 PAs selected based on the EFSA scientific opinion from 2011 [9], which identified key PAs in tea and herbal infusions. The 28 selected PAs include echimidine, heliotrine, lycopsamine, intermedine, erucifoline, senecionine, seneciphylline, monocrotaline, jacobine, senecivernine, retrorsine, europine, lasiocarpine, senkirkine, and their *N*-oxide forms. A total of 274,632 analytical results on PAs in food samples were available, accounting for a total of 19,332 food samples. The number of PAs analyzed per sample ranged between one and 28. To avoid underestimation of the presence of PAs, only those samples with a minimum number of PAs were selected. Special attention was paid to the presence of two additional PAs, riddelliine and riddelliine-*N*-oxide, due to their toxicity. These two PAs were analyzed in 301 samples of tea and herbal infusions, and in all cases, they were reported below the Limit of Quantification (LOQ). Food samples were mainly tea and herbs for infusion, and honey samples A total of 294 samples of food supplements were also available. In addition to honey samples, 825 food samples of animal origin were also part of this data set, with 97% of them having all analyzed PAs as left-censored data. Previous studies demonstrated that the levels of PAs in animal-derived food are much lower than those that can be found in food commodities such as tea and herbal infusions. Among 746 samples of animal origin, only occasional low levels of PAs in milk samples were found, mostly with single PAs in their free base form. Except for two egg samples, PAs were absent in the milk products, eggs, meat, and liver samples analyzed.

On 27 July 2017, the EFSA Panel on Contaminants in the Food Chain (CONTAM) published a statement on the risks to human health related to the presence of pyrrolizidine alkaloids in honey, tea, herbal teas, and dietary supplements [12]. The CONTAM Panel established a new benchmark of 237 μg/kg body weight per day to assess the carcinogenic risks associated with PAs and concluded that exposure to PAs poses a potential risk to human health, especially for people who frequently consume large amounts of tea, herbal teas, and herbal based medicines. The conclusion was also that the younger segment of the population is particularly vulnerable.

In the document of the Standing Committee on Plants, Animals, Food and Feed (PAFF Committee) of 17 April 2018, there is also a reference to PAs [13]. According to the Committee, consideration should be given to setting maximum PA levels for the following foods: tea and herbal infusions, tea for babies and small children, herbal dietary supplements derived from plants containing PAs, and dietary supplements accidentally contaminated with plants containing PAs, honey, and pollen-based dietary supplements.

The toxicity studies on PAs, based on animal experiments [69,72] as well as on human cell lines [70], demonstrated their hepatotoxicity [58,59,61,69,70,72], genotoxicity [62,67,69] and also carcinogenic potential [66]. For these reasons, the EFSA has consistently identified PAs as a serious health risk and has been placing them among the substances requiring careful monitoring in food products. Commission Regulation (EU) 2020/2040 of 11 December 2020 [14] sets maximum levels for PAs in food products, namely for teas and herbal infusions (with lower limits for infants and young children), certain food supplements, pollen and pollen products, dried herbs, borage leaves, and cumin seeds. They refer to the lower limit of the sum of 35 pyrrolizidine alkaloids: intermedine, lycopsamine, intermedine *N*-oxide, lycopsamine *N*-oxide, senecionine, senecivernine, senecionine *N*-oxide, senecivernine *N*-oxide, seneciphylline, *N*-oxide seneciphylline, retrorsine, retrorsine *N*-oxide, echimidine, echimidine *N*-oxide, lasiocarpine, lasiocarpine *N*-oxide, senkirkine, europine, europine *N*-oxide, heliotrine, heliotrine *N*-oxide, indicine, echinatine, rinderine, indicine-*N*-oxide, echinatine-*N*-oxide, rinderine-*N*-oxide, integerrimine, integerrimine-*N*-oxide, heliosupine, heliosupine-*N*-oxide, spartioidine, spartioidine-*N*-oxide, usaramine, usaramine *N*-oxide. Specifically for food supplements, the maximum levels of alkaloids are as follows: Food supplements containing herbal ingredients, including extracts: 400 µg/kg; pollen-based food supplements: 500 µg/kg. In the case of food, the maximum levels of PAs are as follows: Dried herbs and cumin seeds: 400 µg/kg; tea (*Camellia sinensis*) and flavored tea: 150 µg/kg for adults and 75 µg/kg for infants; herbal infusions (dried product) (here are differences in details): 200 µg/kg or 400 µg/kg when concerns herbal teas made from rooibos, anise, lemon balm, chamomile, thyme, peppermint, lemon verbena (dried product) and mixtures exclusively composed of these dried herbs. Limits for any herbal teas for babies are invariably the same as for teas, 75 µg/kg.

In the document Commission Regulation (EU) 2023/915 dated 25 April 2023 these maximum levels for PAs were maintained [57]. Legally, the foodstuffs listed in the Annex to the Regulation placed on the market before 1 July 2022 may be marketed until 31 December 2023. After 1 July 2022, each product covered by the regulation should obligatorily meet the legal requirements in this area.

A European Pharmacopoeia Commission at the European Directorate for the Quality of Medicines (EDQM) published on 1 July 2021 in Supplement 10.6 of the European Pharmacopoeia (Ph. Eur.) the new general chapter “Contaminant pyrrolizidine alkaloids (2.8.26)” [73]. This general chapter, which describes 28 target PAs, allows for the use of any procedure consisting of chromatography coupled with MS/MS or high-resolution MS that meets the validation requirements given in the chapter. This approach was adopted because there is considerable variation in the composition and matrices of the herbal drugs, as well as in the applicable limits, making it difficult to describe all the methods suitable for quantitative analysis of the target PAs [73]. Performance criteria for method validation for PAs are given in the document Commission Implementing Regulation (EU) 2023/2783 dated 14 December 2023 [74] and in the Guidance Document on Performance Criteria for Methods of Analysis for Mycotoxins and Plant Toxins in Food and Feed from the European Union Reference Laboratory (EURL) [75].

Also, the United States Pharmacopeia (USP) has adopted a new Chapter on Pyrrolizidine Alkaloids <1567> Pyrrolizidine Alkaloids as Contaminants in USP-NF 2023 Issue 3 [76].

All the regulators indicate that the presence of PAs in food products can be minimized or prevented by the application of good agricultural and harvest practices. The European Tea and Herbal Infusions Industry has developed a Code of Practice to prevent and reduce pyrrolizidine alkaloid contamination in agricultural commodities used in the manufacture of tea and herbal infusions. This is designed to minimize contamination of materials at the primary producer level [77]. To prevent and reduce PA contamination, management practices such as effective weed control and careful monitoring of animal feed are crucial. It is also important to note that total eradication of PA-containing plants is not feasible or ecologically desirable [3].

## 5. Methods for PA Determination

### 5.1. Sample Preparation for the PAs Containing Materials

PA determination is closely related to the appropriate sample preparation. Efficient PA extraction from biological material is required for a valid determination. Analysis of trace amounts of PAs requires proper sample preparation to increase the PA concentration and remove compounds that interfere with the analysis. Various techniques for PA extraction from medicinal plants are described in the literature [24,31,32]. The applied sample preparation method should efficiently extract the PAs as well as PANOs at the same time. Therefore, the use of polar organic solvents or aqueous solutions is preferred due to the high polarity of PANOs [15,32]. For the PAs and PANOs extraction from different matrices, the most frequently used procedures are solid-liquid extraction (SLE), e.g., sample/methanol [78], and liquid–liquid extraction (LLE), e.g., chloroform/methanol [79]. SLE methods are variations based on maceration or percolation, with additional use of other factors such as sonication, high pressure, or solvent modification. Maceration is the process when the herbal material is continuously soaked with solvent, while during percolation, the solvent flows through the plant material. Generally used solvents are dilute aqueous acids: 0.05 M sulfuric acid, 0.15 M hydrochloric acid solution, 0.5% formic acid, and polar organic solvents, e.g., acidified methanol or acetonitrile [31]. Organic solvents such as chloroform and dichloromethane may also be used, but this approach is problematic because it requires additional steps. The extract should be dissolved in an aqueous acidic solution and washed with a non-polar solvent, e.g., chloroform, to remove less polar material (such as fats, waxes, and terpenes). The addition of ammonia makes the solution strongly basic, and the PAs are extracted back into an organic solvent. Repetition of this process provides extracts clean enough for GC-MS [32]. PANOs are less soluble in relatively non-polar solvents, and to determine the entire profile of PA free bases and *N*-oxides, in the first step, PA free bases are extracted alone, and in the second step, after reduction of the *N*-oxides, the total content of PAs is obtained. The proportion of the *N*-oxides can then be determined by calculating the difference between these two measurements [22,80].

Sample purification is often a necessary step, and solid-phase extraction (SPE) is then used, e.g., solid-phase extraction cartridges (SPE) [81]. There are two main types of SPE: strong cation exchange (SCX) SPE and reversed-phase SPE. SCX-SPE is a silica-based benzenesulfonic acid-based filler. Its negatively charged sulfonic acid group has a strong cation exchange capacity, and the benzene ring has a certain hydrophobic retention. SCX extracts positively charged basic compounds, such as amines. Reversed-phase SPE is a slightly selective separation technique. Reversed-phase sorbents, mainly based on octadecylsilane ligands (C18), can retain most molecules with hydrophobic character, making them very useful for extracting analytes that are very diverse in structure within the same sample [82]. Mixed-mode sorbents (a combination of reversed-phase and cation-exchange interactions) are also used in SPE cartridges. For this purpose, SPE is commonly used for the purification of PAs/PANOs from food samples [31]. The technique that is very often used for the determination of PAs/PANOs in food samples is QuEChERS (acronym of “Quick, Easy, Cheap, Effective Rugged and Safe”). This procedure assumes the simultaneous extraction and purification of the samples and is suitable for extracting a large number of compounds. This procedure is miniaturized and can be successfully applied to the analysis of 21 PAs/PANOs in oregano, significantly reducing the amount of reagents used by ten times in comparison to the classic methodology [83,84]. A broad range of extraction procedures to determine PAs/PANOs in dried plants and food supplements was presented in the review [42].

### 5.2. LC-and GC-Methods for PAs Analysis

Following the successful extraction, sample solutions can be analyzed using chromatographic separation techniques HPLC and GC coupled with various types of detection.

The structural diversity of PAs and PANOs is a challenge for analysts. According to EFSA requirements, the total sum and individual amounts of PAs should be determined in plant material [11]. Fast qualitative tests to measure total PA content, instrumental methods to determine the PA profile in samples, and sensitive quantification of these alkaloids are needed. Spectroscopic techniques can be utilized in such studies. However, these methods are not sensitive enough to determine trace amounts of PAs. Ultraviolet–visible spectroscopy (UV–Vis) methods and colorimetry are used for PA detection, rather than for quantitative measurements [31]. Nuclear magnetic resonance (NMR) spectroscopy methods were applied to PA analysis for structural identification and identity confirmation of novel PAs [85]. The use of immunoassays for the determination of PAs is rare, but the classical enzyme-linked immunosorbent assay (ELISA) can be used for the analysis of PAs [86]. The technique is hampered by the presence of some cross-reactivity, preventing easy detection of target PAs. The most popular and useful techniques are chromatographic techniques. Preparative HPLC and TLC can be used for PA isolation [32]. TLC, GC, and HPLC methods for PA analysis are also used; however, these methods are suitable for working with materials containing quite a high PA content. A recent review of the analysis of PAs in medicinal plants refers to plants from genera that naturally produce these alkaloids: *Tussilago* (coltsfoot), *Symphytum* (comfrey), *Senecio*, *Petasites* (butterburs), *Lithospermum* (gromwell), *Heliotropium* (bloodstone), *Cynoglossum*, *Borago*, *Brachyglottis Anchusa*, and *Alkanna* [24]. To determine trace amounts of PAs, methods based on mass spectroscopy should be used, which is recommended by the Ph. Eur. [73].

### 5.3. Electrochemical Methods for PAs Analysis

Alkaloid detection methods that use detection techniques such as ELISA, and MS/MS are laborious, time-consuming, expensive, and require the use of complex equipment and staff training. Currently, fast, simple, and accurate methods are being developed to monitor alkaloids in real samples. These benefits are related to the widespread use of electrochemical sensors modified with various materials [87,88,89]. Electrochemical detection was found to be favorable because it is easy to use, affordable, rapid, and highly sensitive, and the analysis can be performed on-site [43,90]. Various electrochemical techniques can be applied for quantitative analysis, mainly cyclic or stripping voltammetry (CV), differential pulse voltammetry (DPV), and more recently, electrochemical impedance spectroscopy (EIS). Using electrochemical biosensors, researchers can identify specific analytes present in biological samples and convert biochemical signals into electrical signals, simplifying quantification [87,88,89]. Electrochemical sensors are also convenient because they can be easily miniaturized, demonstrate excellent sensitivity, and are simple to construct and use. Electrodes are used in detection and are mainly composed of carbonaceous materials such as glassy carbon (GCE), pyrolytic graphite (HOPG), screen-printed carbon electrodes (SPCE), and even still mercury electrodes [91]. By modifying the surfaces of these electrodes with various materials, good selectivity can be achieved [92]. Improvement of sensitivity and resolution is obtained by using the DPV compared to CV, which is important, especially in detecting traces of alkaloids. EIS is very useful in studying surface processes, kinetics, and mechanisms of alkaloid reactions. Recently, the surfaces of electrodes have been additionally decorated with nanoparticulate materials to increase the sensitivity and selectivity of the sensor. Nanomaterials are selected so that their physical and chemical properties match the detected analyte as much as possible. Particular attention is paid then to their chemical composition, crystal structure, orientation of the crystallographic axis, morphology, and dimensions of the nanoparticles. Inorganic and organic nanomaterials used for electrode modification include carbon and metallic nanoparticles, polymer materials, and others [43,90]. Nanomaterials have different shapes, such as nanoflowers, nanowires, nanorods, or nanofibers. Due to their specific surface, high conductivity, and electrocatalytic properties, they are widely used to improve detection limits and specificity. The modified electrodes are useful in testing alkaloids in biological, pharmacological, and agri-food matrices. The determination of alkaloids in biological samples: serum, urine, blood, etc., is crucial in forensics and clinical applications [43,90]. It is important to determine alkaloids in human body fluids, especially when alkaloid poisoning is suspected. The analysis must be performed immediately, and the analyte has to be identified at low concentrations [93,94,95]. Nanomaterial-modified electrodes are also used for the determination of various alkaloids in pharmaceutical samples because the analytical procedures are simple, fast, and accurate. Trace amounts of various alkaloids can be determined individually or simultaneously. To our knowledge, among the very large number of sensors for the determination of alkaloids, only a few sensors designed specifically for the determination of PAs can be found in the literature.

Erdem and coworkers [96] developed a simple and inexpensive electrochemical test based on a single-use sensor for the quantification of senecionine (SEN) in food. SEN was immobilized on the surface of a pencil graphite electrode. The SEN oxidation signal was used to evaluate the sensor using differential pulse voltammetry (DPV). The selectivity of the sensor was also checked in the presence of other similar PAs such as intermedine, lycopsamine, and heliotrine. The detection limit was 5.45 μg/mL. Electrochemical detection of SEN had high sensitivity and good selectivity. The sensor was also tested by examining its use in flour and herbal tea products.

Yang and coworkers [97] developed a visual, easy-to-use, and cost-effective mesoporous silica-based electrochemiluminescence (MPS-ECL) sensor for point-of-care (POC) testing of PAs. ECL activity was found to depend on the PA structure. The intensity of ECL also varies for different PAs in order: monocrotaline ˃ senecionine *N*-oxide ˃ retrorsine ˃ senkirkine. The POC sensors had excellent linearity, low detection limits (0.02 µM–0.07 µM), and good recovery, indicating good accuracy and practicality. The portable and low-cost sensor is user-friendly and can be used to test PAs in drugs, food products, and clinical samples, which shows promise in the preliminary assessment of PA-induced health risks. The sensor is repeatable and temperature stable and was used to perform on-site PA screening in milk, tea, herbal medicines, and human serum samples.

### 5.4. LC-MS and GC-MS Methods for PAs Analysis

PA contamination of herbal products is usually at low levels, so sensitive analytical methods based on mass spectrometry (MS), such as liquid chromatography-mass spectrometry (LC-MS) and gas chromatography-mass spectrometry (GC-MS), are required for their determination. LC-MS is now the preferred method for the determination of PAs [31,32]. LC-MS and LC-MS/MS methods have become the most popular approaches to the identification and quantification of PAs as they combine reliability and high sensitivity with ease of sample preparation. A range of mass spectrometer types can be used, including single quadrupoles (MS), ion traps (IT), triple quadrupoles (QqQ; MS/MS), and time of flight (ToF) instruments. The ionization method is primarily electrospray ionization (ESI). Atmospheric pressure chemical ionization (APCI) can also be applied, but it is less sensitive. High sensitivity is provided by the positive electrospray ionization modes (ESI +) [31]. In LC-MS methods, the experiment can be run in single-ion monitoring (SIM) or scan mode. The detection and quantification of PAs using SIM were successfully performed [98]. In LC-MS/MS methods, collision-induced dissociation (CID) of the PA molecular ion provides fragment ions that can be used in selected reaction monitoring (SRM) or multiple reaction monitoring (MRM). Applying MRM detection that uses the transition from the molecular ion to the specific fragments of the molecule, the highest sensitivity and specificity in PA analysis could be obtained [33,34,35,36,37,38,39,40,41]. SIM detection on MS/MS also was applied for PAs analysis [99]. SRM provided greater sensitivity and selectivity than high-resolution SIM on a single quadrupole [100]. High-resolution mass spectrometry (HRMS) is the latest approach to the analysis of complex sample matrices, such as plant-based products. The increased resolution of HRMS instrumentation enables the resolution of isotope distributions and the generation of fragmentation paths. It uses mass analyzers such as ToF and Orbitrap, which have high mass resolving power that can be used to generate high-quality results. Nevertheless, HRMS instrumentation does not replace the standard low-resolution mass spectrometers found in many research laboratories [101]. The identification and quantification of 25 PAs and *N*-oxides using LC-Q-ToF/MS was performed [102]. A new type of mass spectrometer, the Orbitrap, has brought a significant change to high-resolution mass spectrometry. PAs in botanical samples and PA environmental degradation products were examined using the Orbitrap MS [103,104,105,106,107]. GC-MS analysis of PAs was performed on a single quadrupole spectrometer and was limited to single PA [108], qualitative analysis [109], and the analysis of volatile PAs [110].

An overview of the methods for PA determination in various food products is given in Table 4.

## 6. Safety Ensuring, Prevention, and Market Control

The European Union (EU) has a range of tools to ensure food safety, including the Rapid Alert System for Food and Feed (RASFF). It has been set up for exchanging information between member countries and supports the rapid response of food safety authorities to public health. It is effective at every stage of the food chain. An interactive, searchable online database called RASFF Window provides public access to summary information about the most recently transmitted RASFF notifications and allows searching for information on any notification issued in the past (currently limited to 2020 and later) [121]. The database was searched on 28 April 2024, using the keyword “pyrrolizidine”, and 136 records were collected in Table 5. The permissible PA level was exceeded in the products mentioned.

## 7. Conclusions

The growing interest in plant-based medicinal products and dietary supplements should not be associated with the misconception that these products are inherently safe and free of side effects. PAs are a class of natural toxins that draw significant attention due to their presence in honey and medicinal plants. PAs are proven to be carcinogenic, genotoxic, and hepatotoxic. Metabolic activation of PAs leads to the formation of adducts with DNA, which is considered to be the main cause of the carcinogenic effects of PAs. The toxic nature of PAs poses a potential risk to human health. Human consumption of PAs from food and herbal medicinal products has likely remained stable over recent years, but new, sensitive analytical methods can now detect very low levels of PAs. However, once a problem is identified, regulatory action to mitigate it should be considered. Therefore, the development and validation of sensitive analytical methods, especially those based on LC-MS, are of great importance to ensure consumer safety and improve public health. In this review, we introduced sensitive and selective analytical methods for the determination of PAs in various materials. Reporting new cases of contamination is important to ensure the safety of herbal products.

## 8. Future Directions

Despite significant progress in the determination of PAs in various matrices, the structural diversity and chemical properties of PAs present unique challenges to analysts. The continuous development of analytical methods is essential to improving the detection and quantification of PAs. From a future perspective, the determination of PA in plant and bee products should be a mandatory preventive step during production. The development of convenient, fast, and sensitive electrochemical sensors can be an alternative to complex mass spectrometry-based methods.

## Figures and Tables

**Table 1:** The chemical structures of PAs listed in Annex 1 to the Commission Regulation (EU) 2023/915. Twenty-one PAs whose concentration should be monitored (**n**-numbers) and an additional 14 PAs (**na** numbers) who are known to co-elute with one or more of the requiring investigation 21 PAs.

No.	Name	Alkaloid Chemical Structure	Chemical Structure of Corresponding *N*-Oxide
**1, 2**	Echimidine, echimidine-*N*-oxide	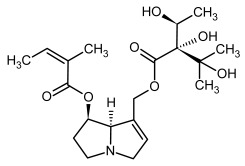	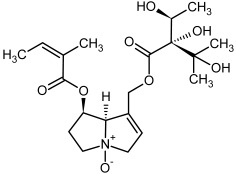
	Possible co-elution of **1** and **2** with, respectively:		
**1a, 2a**	Heliosupine, heliosupine-*N*-oxide	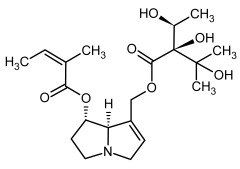	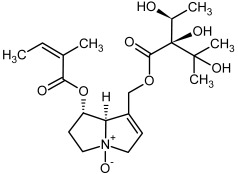
**3, 4**	Heliotrine, heliotrine-*N*-oxide	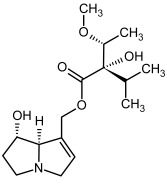	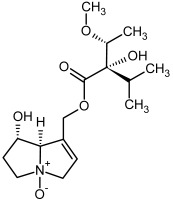
**5, 6**	Intermedine, intermedine-*N*-oxide	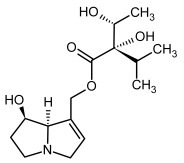	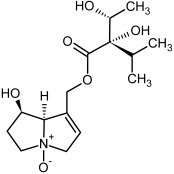
**7, 8**	Lycopsamine, lycopsamine-*N*-oxide	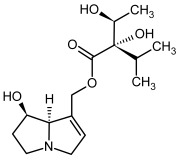	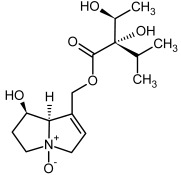
	Possible co-elution of **5**, **6**, **7**, and **8** with, respectively:		
**3a, 4a**	Indicine, indicine-*N*-oxide	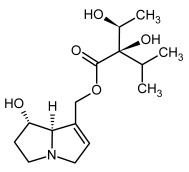	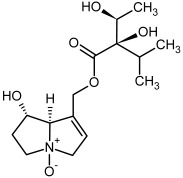
**5a, 6a**	Echinatine, echinatine-*N*-oxide	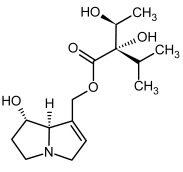	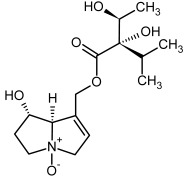
**7a, 8a**	Rinderine, rinderine-*N*-oxide	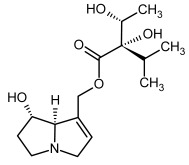	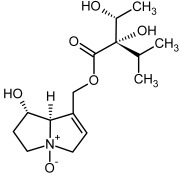
**9, 10**	Retrorsine, retrorsine-*N*-oxide	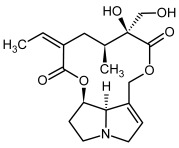	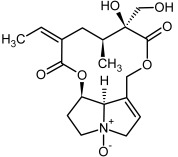
	Possible co-elution of **9** and **10** with, respectively:		
**9a, 10a**	Usaramine, usaramine-*N*-oxide	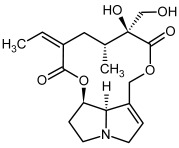	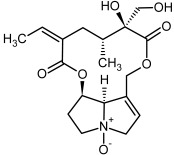
**11, 12**	Senecionine, sencionine-*N*-oxide	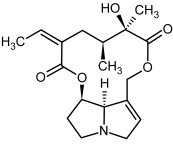	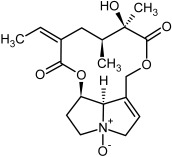
**13, 14**	Senecivernine, senecivernine-*N*-oxide	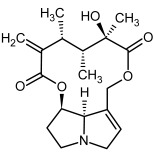	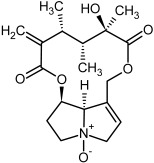
	Possible co-elution of **11**, **12**, **13** and **14** with:		
**11a, 12a**	Integerrimine, integerrimine-*N*-oxide	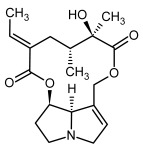	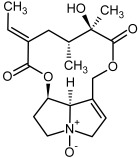
**15, 16**	Seneciphylline, seneciphylline-*N*-oxide	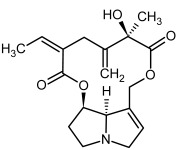	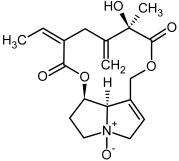
	Possible co-elution of **15** and **16** with, respectively:		
**13a, 14a**	Spartioidine, spartioidine N-oxide	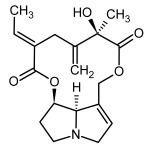	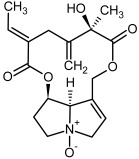
**17, 18**	Europine, europine-*N*-oxide	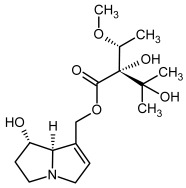	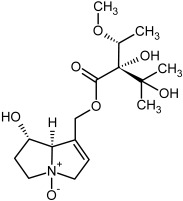
**19, 20**	Lasiocarpine, lasiocarpine-*N*-oxide	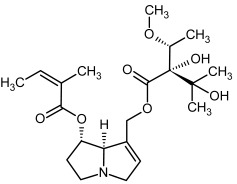	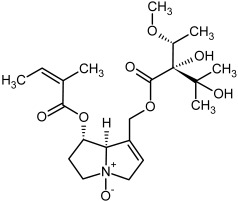
**21**	Senkirkine	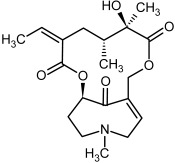	

**Table 2:** Chemical structures of PAs not listed in Table 1 (nb numbers), which, according to the EFSA opinion, should also be monitored in food and feed [8].

No.	Name	Alkaloid Chemical Structure	Chemical Structure of Corresponding N-Oxide
**1b, 2b**	Erucifoline, erucifoline-*N*-oxide	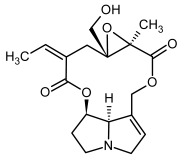	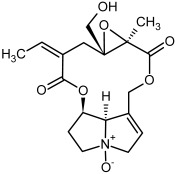
**3b, 4b**	Monocrotaline, monocrotaline-*N*-oxide	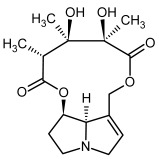	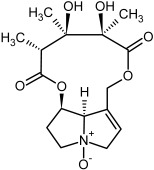
**5b, 6b**	Trichodesmine, trichodesmine-*N*-oxide	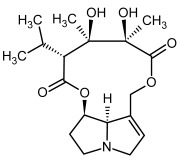	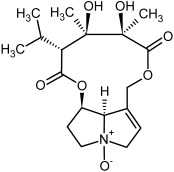
**7b, 8b**	Jacobine, jacobine-*N*-oxide	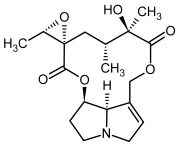	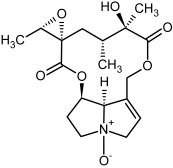
**9b, 10b**	Jaconine, jaconine-*N*-oxide	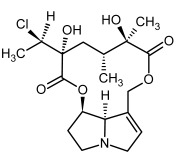	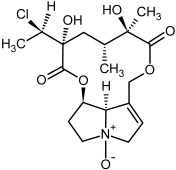

**Table 3:** LD_50_ values of the most important PAs.

	In Silico Oral/Rat LD_50_ (mg/kg Body Weight)	In Vivo Intraperitoneal LD_50_ (mg/kg Body Weight)	Species
Compound/Ref.:	[70]	[2]	
Echimidine	616	200	rat, male
Echinatine	250	350	rat, male
Europine	-	>1000	rat, male
Heleurine	616	140	rat, male
Heliosupine	708	60	rat, male
Heliotridine	-	1500	rat, male
Heliotrine	56	296	rat, male
Heliotrine	-	478	rat, female
Indicine	264	>1000	rat, male
Intermedine	264	1500	rat, male
Jacobine	461	138	rat, female
Jaconine	-	168	rat, female
Lasiocarpine	555	77	rat, male
Lasiocarpine	-	79	rat, female
Lycopsamine	239	1500	rat, male
Monocrotaline	-	154	mouse
Monocrotaline	731	109	rat, male
Monocrotaline	-	230	rat, female
Otonecine	467	-	-
Platyphylline	443	252	rat, male
Retronecine	242	-	-
Retrorsine	320	34–38	rat, male
Retrorsine	-	153	rat, female
Riddelliine	616	80	rat, male
Rinderine	486	550	rat, male
Senecionine	127	50	rat, male
Seneciphylline	264	77	rat, male
Seneciphylline	-	83	rat, female
Senecivernine	592	-	-
Senkirkine	275	220	rat, male
Spectabiline		50	rat, male
Supinine	215	450	rat, male
Symphytine	-	130	rat, male
Usaramine	264	-	-
Trichodesmine	324	-	-

**Table 4:** Analytical separation and detection techniques for the determination of PAs in herbal material.

Sample Type	PAs	Separation Technique	Chromatographic Conditions		Detection	LOD/LOQ	Ref.
			Column	Elution			
*Senecio brasiliensis* beehive pollen honey	senecionine senecionine *N*-oxide retrorsine *N*-oxide	HPLC	C_18_ 100 mm × 3.0 mm, 3.5 µm (manufacturer undefined)	Mobile phase A: water with 0.1% formic acid Mobile phase B: acetonitrile with 0.1% formic acid Gradient: 98% A from 0 to 2.0 min, 85% A from 2.0 to 5.0 min, 50% A from 5.0 to 8.0 min, 10% A from 8.0 to 9.0 min, 98% A from 9.0 to 11.0 min.	Q-TRAP; MS/MS; Mode: ESI + MRM	-	[34]
bee-collected pollen teas herbal infusions	acetyllycopsamine echimidine group europine heliotrine intermedine lasiocarpine lycopsamine group retrorsine group senecionine group seneciphylline group senkirkine trichodesmine	UHPLC	Accu-core^TM^ RP-MS (Thermo Scientific, Waltham, MA, USA) 100 mm × 2.1 mm, 2.6 μm	Mobile phase A: 0.1% formic acid in water Mobile phase B: methanol/acetonitrile 1:1 (*v*/*v*). Gradient: from 3% to 4% B (0–1 min), from 4% to 17% B (1–6 min), 17% B held for 2 min, from 17% to 44% B (8–10.5 min), from 44% to 95% B (in 0.1 min), 95% B held for 1 min, from 95% to 3% B in 0.1 min re-equilibration to 3% B for 4 min.	QqQ; MS/MS; Mode: ESI + MRM	LOD: 2.4–5.3 ng/g LOQ: 4.0–9.0 ng/g LOD: 0.04–0.08 ng/mL LOQ: 0.07–0.14 ng/mL	[111]
oregano	intermedine europine lycopsamine europine *N*-oxide intermedine *N*-oxide lycopsamine *N*-oxide retrorsine retrorsine *N*-oxide seneciphylline heliotrine heliotrine *N*-oxide senecivernine senecionine seneciphylline *N*-oxide senecivernine *N*-oxide senecionine *N*-oxide echimidine echimidine *N*-oxide lasiocarpine lasiocarpine *N*-oxide senkirkine	UHPLC	Luna Omega Polar C_18_ (Phenomenex, Torrance, CA, USA), 100 mm × 2.1 mm, 1.6 µm	Mobile phase A: 0.2% formic acid and 5 mM ammonium acetate in water Mobile phase B: 10 mM ammonium acetate in methanol Gradient: 5% B (0–0.5 min), 5–50% B (0.5–7 min), 50% B (7–7.5 min), 50–100% B (7.5–11 min), 100% B (11–12 min), 100–5% B (12–14 min). re-equilibrated with the initial composition for 1 min.	IT; MS/MS; Mode: ESI + TIC	LOD: 0.1–7.5 ng/g LOQ: 0.5–25.0 ng/g	[83]
black tea, green tea dark tea *Chrysanthemum* weed	heliotrine heliotrine-*N*-oxide retrorsine retrorsine-*N*-oxide senecionine senecionine-*N*-oxide jacobine jacobine-*N*-oxide intermedine intermedine-*N*-oxide seneciphylline seneciphylline-*N*-oxide europine senkirkine	UHPLC	Waters Acquity UPLC HSS T3 (Waters, Milford, MA, USA) 100 mm × 2.1 mm, 1.8 μm	Mobile phase A: methanol buffered with 0.1% formic acid and 1 mM ammonium formate. Mobile phase B: Water buffered with 0.1% formic acid and 1 mM ammonium formate. Gradient: MPB was applied: 0–1 min at 90%, 1–4 min from 90% to 40%, 4–7 min from 40% to 30%, 7–7.1 min from 30% to 2%, 7.1–11 min at 2%, 11–11.1 from 2% to 90%, held for 2.9 min before the next run.	QqQ; MS/MS; Mode: ESI + MRM	LOD: 0.001–0.4 ng/g LOQ: 1–5 ng/g	[112]
honey	echimidine intermedine lycopsamine retrorsine retrorsine *N*-oxide senecionine senecionine *N*-oxide echimidine *N*-oxide erucifoline erucifoline *N*-oxide europine europine *N*-oxide heliotrine heliotrine *N*-oxide intermedine intermedine *N*-oxide jacobine jacobine *N*-oxide lasiocarpine lasiocarpine *N*-oxide lycopsamine lycopsamine *N*-oxide monocrotaline monocrotaline *N*-oxide seneciphylline seneciphylline *N*-oxide senkirkine trichodesmine	UHPLC	Waters Acquity UPLC BEH C18 (Waters, Milford, MA, USA) 100 mm × 2.1 mm, 1.7 μm	Mobile phase A: 6.5 mM ammonium hydroxide in water Mobile phase B: 6.5 mM ammonium hydroxide in acetonitrile Gradient: 0 to 2 min: 0% B; 2 to 10 min: 0 to 50% B, maintained to 2 min; 12 to 14 min: 50 to 100% B, maintained to 16 min; 16 to 19 min: 100 to 0% B, maintained to 23 min.	QTOF-MS/MS; Mode: ESI +	LOD: 1–7 ng/g LOQ: 10–20 ng/g	[35]
*Tussilago farfara* *Lithospermum erythrorhizon*	echimidine echimidine *N*-oxide erucifoline erucifoline *N*-oxide europine europine *N*-oxide heliotrine heliotrine *N*-oxide intermedine intermedine *N*-oxide jacobine jacobine *N*-oxide lasiocarpine lasiocarpine *N*-oxide lycopsamine lycopsamine *N*-oxide monocrotaline monocrotaline *N*-oxide retrorsine retrorsine *N*-oxide senecionine senecionine *N*-oxide seneciphylline	HPLC	Shim-pack GIST-C18 (Shimadzu Corporation, Kyoto, Japan) 150 mm × 2.1 mm, 2 μm	Mobile phase A: 0.1% formic acid in 5 mM ammonium formate Mobile phase B: 0.1% formic acid plus 5 mM ammonium formate in 100% methanol Gradient: 1.5 min, 1% B; 1.5–3.0 min, 1–15% B; 3.0–18.0 min, 15–30% B; 18.0–19.0 min, from 30 to 95% B 19.0–21.0 min, 95% B; 21.1 min, 1% B.	QqQ; MS/MS Mode: ESI + MRM	LOD: 0.5–1.7 ng/g LOQ: 1.7–6.4 ng/g	[36]
*Sorghum* oregano mixed herbal tea	echimidine echinatine erucifoline europine heliotrine indicine intermedine jacobine lasiocarpine lycopsamine monocrotaline retronecine retrorsine senecionine seneciphylline senecivernine senkirkine trichodesmine echimidine *N*-oxide echinatine *N*-oxide erucifoline *N*-oxide europine *N*-oxide heliotrine *N*-oxide indicine *N*-oxide intermedine *N*-oxide jacobine *N*-oxide lasiocarpine *N*-oxide lycopsamine *N*-oxide monocrotaline *N*-oxide retrorsine *N*-oxide senecionine *N*-oxide seneciphylline *N*-oxide senecivernine *N*-oxide	UHPLC	Waters Acquity UPLC® BEH Amide (Waters, Milford, MA, USA) 100 mm × 2.1 mm; 1.7 μm	Mobile phase A: water with ammonium formate 5 mM Mobile phase B: acetonitrile: water 95:5, *v*/*v*, with formic acid (0.1%, *v*/*v*). Gradient: 1.5 min, 1% B; 1.5–3.0 min, 1–15% B; 3.0–18.0 min, 15–30% B; 18.0–19.0 min, from 30 to 95% B; 19.0–21.0 min 95% B; 21.1 min, return to 1% B.	Q-TRAP; MS/MS Mode: ESI + MRM	LOD: - LOQ: 0.5–10 ng/g	[33]
tea	echimedine heliotrine lasiocarpine lycopsamine monocrotaline monocrotaline *N*-oxide retrorsine-*N*-oxide retrorsine senecionine-*N*-oxide senecionine seneciphylline *N*-oxide seneciphylline senkirkine trichodesmine europine-*N*-oxide intermedine jacobine europine jacobine *N*-oxide lasiocarpine *N*-oxide heliotrine *N*-oxide	UPLC	Waters X-Bridge (Waters, Milford, MA, USA) C18, 100 mm × 2.1 mm, 3.5 µm	Mobile Phase A: 5 mM ammonium formate and 0.1% formic acid Mobile Phase B: 95% methanol with 5 mM ammonium formate and 0.1% formic acid Gradient: 5% B for 0.5 min, increasing B from 5% to 30% for 6.5 min, from 30% to 95% for 4 min and then holding for 2 min, decreasing to 5% for 0.1 min, and finally holding for 1.9 min	QqQ; MS/MS Mode: ESI + MRM	LOD: 0.1–3.0 ng/g LOQ: 0.3–9.0 ng/g	[113]
plant material tea		SFC	CHIRALPAK®, IG-3/SFC, (Daicel Chiral Technologies, Shanghai, China) 100 mm × 3 mm, 3 µm,	Mobile Phase A: CO_2_ Mobile Phase B: 50 mM Ammonium formate in methanol Mobile Phase C: Methanol Mobile Phase D: 0.1% Formic acid	QqQ; MS/MS Mode: ESI +	LOQ: 2–200 ng/g	[114]
plant based food herbal tea	erucifoline *N*-oxide europine europine *N*-oxide, jacobine, retrorsine, retrorsine *N*-oxide, seneciphylline *N*-oxide, senecivernine *N*-oxide trichodesmine	UHPLC	Waters Acquity UPLC® BEH C18 (Waters, Milford, MA, USA) 100 mm × 2.1 mm, 1.7 μm	Mobile phase A: water with 0.1% ammonia Mobile phase B: acetonitrile. Gradient: Starting at 5% of phase B, kept for 1 min, rising to 15% till 2 min before a new isocratic separation for 1 min, increasing to 20% (from 3 to 5 min), 25% (from 5 to 6 min), 50% (from 6 to 9 min) and 95% (from 9 to 10 min).	QqQ; MS/MS Mode: ESI + MRM	LOD: - LOQ: 0.5–1 ng/g	[115]
maize	total	HPLC	Synergy Max-RP 80 Å (Phenomenex, Aschaffenburg, Germany) 150 mm × 2.1 mm, 4 μm,	Mobile phase A: 0.3% formic acid in water Mobile phase B: 0.3% formic acid in acetonitrile) Gradient: 2 min (95% A), 14 min (95–40% A), 15 min (40–0% A), 18 min (0% A), 19 min (95% A), 30 min (reequilibration 95% A).	Q-TRAP; MS/MS Mode: ESI + MRM	-	[81]
*Gynura japonica* milk	senecionine, seneciphylline, senkirkine, retrorsine	DART-MS HPLC-MS	Waters Acquity UHPLC BEH C18 (Waters, Milford, MA, USA) 2.1 mm × 100 mm, 1.7 μm	Mobile phase A: water with 0.1% formic acid Mobile phase B: acetonitrile Gradient: 0–3 min, B 3%; 3–6 min, B 3–10%; 6–8 min, B 10–100%; 8–10 min, B 100–3%; 10–15 min, Re-equilibration, B 3%.	IT; MS/MS Mode: ESI +	LOD: 0.55–0.85 ng/mL LOQ: 1.83–2.82 ng/mL	[116]
herbal food supplements	monocrotaline, intermedine, monocrotaline *N*-oxide, indicine, lycopsamine, europine, europine *N*-oxide, indicine *N*-oxide, riddelliine, junction, riddelline *N*-oxide, trichodesmine, retrorsine, retrorsine *N*-oxide, heliotrine, seneciphylline, heliotrine *N*-oxide, seneciphylline *N*-oxide, integerrimine, senecionine, senecionine *N*-oxide, senkirkine, echimidine, lasiocarpine, lasiocarpine *N*-oxide	UHPLC	Agilent Poroshell 120 EC-C18 (Agilent Technologies, Palo Alto, CA, USA) 2.1 mm × 150 mm, 2.7 μm	Mobile phase A: water with 0.1% formic acid Mobile phase B: acetonitrile with 0.1% formic acid Gradient: 0–23 min, 3–4% B; 23–45 min, 4–15% B; 45–55 min, 15–25% B 55–57 min 25–100% B. 3 min wash-100% B 5 min Re-equilibration 3% B	QToF-MS/MS Mode: ESI + TIC	LOD: 0.05–5 ng/mL LOQ: -	[78]
black tea, green tea mixed tea flavoured tea herbal tea (chamomile, sage linden, fennel, rosehips) culinary herb samples (thyme, peppermint)	29 pyrrolizidine alkaloids	UHPLC	Agilent Poroshell 120 EC-C18 (Agilent Technologies, Palo Alto, CA, USA) 2.1 mm × 150 mm, 2.7 μm	Mobile phase A: 0.1% formic acid in water Mobile phase B: 0.1% formic acid in acetonitrile Gradient: 0–23 min, 3–4% B; 23–45 min, 4–15% B; 45–55 min, 15–25% B 55–57 min to 100% B. 3 min wash with 100% B 5 min reequilibration with 3% B.	Q-TOF/MS Mode: ESI + Product Ion	LOD: 0.105–0.867 ng/g LOQ: 0.357–2.890 ng/g	[102]
milk	51 pyrrolizidine alkaloids	HPLC	Kinetex EVO C18 (Phenomenex, Torrance, CA, USA), 100 mm × 2.1 mm, 2.6 μm. Kinetex EVO C18 (Phenomenex, Torrance, CA, USA), 150 mm × 2.1 mm, 5 μm.	acidic conditions: Mobile phase A: water with ammonium formate and formic acid 5 mmol/L Mobile phase B: acetonitrile/water (95/5, *v*/*v*), 26.5 mmol/L alkaline conditions: Mobile phase A: ammonium carbonate in water 10 mmoL/L Mobile phase B: acetonitrile	QqQ; MS/MS Mode: ESI + MRM	LOD: 0.005–0.054 ng/g LOQ: 0.009–0.123 ng/g	[117]
black tea, peppermint tea, mixed herbal tea, valerian herbal supplement, alfalfa, hay, sunflower expeller, bovine compound feed	43 pyrrolizidine alkaloids	UPLC	alkaline conditions: Waters Acquity UPLC BEH C18 (Waters, Milford, MA, USA) 2.1 mm × 150 mm, 1.7 μm acidic conditions: Waters Acquity UPLC CSH C18 (Waters, Milford, MA, USA) 2.1 mm × 150 mm, 1.7 μm	alkaline conditions: Mobile phase A: 10 mM ammonium carbonate in water, pH 9 Mobile phase B: acetonitrile acidic conditions: Mobile phase A: 0.1% formic acid in water Mobile phase B: acetonitrile	QqQ; MS/MS Mode: ESI + MRM	LOD: - LOQ: 10 ng/g	[118]
honey	retronecine	GC	Zebron ZB-5MS (Phenomenex, Torrance, CA, USA), 30 m × 0.25 mm; film 0.25 μm	-	Q; MS Mode: positive SIM	LOD: 2 ng/g LOQ: 6 ng/g	[108]
honey	echimidine heliotrine intermedine lasiocarpine lycopsamine retrorsine seneciphylline senecionine senkirkine	UHPLC	Supelco Analytical C8 (Supelco, Bellefonte, PA, USA), 150 mm × 3 mm, 2.7 μm	Mobile phase A: 0.5% formic acid in water Mobile phase B: acetonitrile	Q; MS Mode: ESI + SIM	LOD: - LOQ: 0.08–4.3 ng/g	[98]
honey	lycopsamine senecionine senecionine *N*-oxide heliosupine echimidine	HPLC	Phenomenex Synergi hydro-RP C18, (Phenomenex, Torrance, CA, USA), 100 mm × 30 mm, 2.5 μm	Mobile phase A: 0.1% formic acid in water Mobile phase B: 0.1% formic acid in acetonitrile	QqQ; MS/MS Mode: ESI + SIM	LOD: 0.45–0.67 ng/mL LOQ: 1.21–1.79 ng/mL	[99]
*Tussilago farfara*	senecionine senkirkine	HPLC-DAD	Waters Xterra C18 (Waters, Milford, MA, USA) 3.9 mm × 150 mm, 5 μm	Mobile phase A: 0.1% formic acid in 20 mM NH_4_CH_3_CO_2_; Mobile phase B: 0.1% formic acid in acetonitrile	Q; MS Mode: ESI + SIM	LOD: 0.26/1.32 ng/g LOQ: 1.04/5.29 ng/g	[119]
*Pardoglossum cheirifolium*	9 pyrrolizidine alkaloids	GC	Restek Rxi-1 ms (Restek, Bellefonte, PA, USA), 30 m × 0.25 mm; film 0.25 μm	-	Q; MS TIC	LOD: - LOQ: -	[110]
tea, potato, beans.	15 pyrrolizidine alkaloids	UHPLC	Waters Acquity HSS T3 (Waters, Milford, MA, USA) 2.1 mm × 50 mm, 1.7 μm	Mobile phase A: water with 0.1 % formic acid and ammonium formate 4 mmol/L. Mobile phase B: methanol	Q-Orbitrap-MS/MS Mode: ESI + HRMS	LOD: 1.18–13.28 ng/g LOQ: -	[104]
herbal infusions, rooibos, anise, lemon balm, chamomile, thyme, peppermint, lemon verbena, mixtures of teas of *Camellia sinensis*, flavoured teas, 73 plant-based food supplements (formulated as solid forms, infusions, and sirups).	118 pyrrolizidine alkaloids	UHPLC	Phenomenex Luna Omega Polar C_18_ (Phenomenex, Torrance, CA, USA), 2.1 mm × 100 mm, 1.6 μm	Mobile phase A: 0.1% formic acid in water Mobile phase B: 0.1% formic acid in acetonitrile	Q-Orbitrap-HRMS/MS Mode: HESI-II + Full MS/dd-MS^2^	LOD: 0–1.5 ng/mL LOQ: 0.1–2.1 ng/g in solids; 1–12 ng/g in infusions	[105]
Common heliotrope (*Heliotropium europaeum*) *Heliotropium* *popovii* Chamomile (*Matricaria recutita*)	35 pyrrolizidine alkaloids	UHPLC	Waters Acquity UPLC BEH C18 (Waters, Milford, MA, USA) 150 mm × 2.1 mm, 1.7 μm	Mobile phase A: 10 mM ammonium carbonate in water, pH 9 Mobile phase B: acetonitrile	Q-Orbitrap-MS/MS Mode: HESI-II + Full MS Scan	LOD: - LOQ: -	[106]
rooibos, chamomile, red tea, black tea, green tea, white tea, linden, horsetail, mixture of herbs.	28 pyrrolizidine alkaloids	HPLC	C18	Mobile phase A: 0.1% formic acid in water Mobile phase B: 0.1% formic acid in acetonitrile	Q-Orbitrap-MS/MS Mode: ESI + HRMS	LOD: - LOQ: 5 ng/g	[107]
*Crotalaria* (*Fabaceae*) species	45 pyrrolizidine alkaloids	UHPLC	Hypersil GOLD aQ C18 (Thermo Scientific, Waltham, MA, USA) 100 mm × 2.1 mm, 1.9 μm	Mobile phase A: formic acid in water Mobile phase B: formic acid in acetonitrile (various formic acid concentrations: 0.05, 0.2, and 0.35% *v*/*v*)	Orbitrap-MS Mode: HESI-II + Full MS Scan	LOD: 0.05 ng/mL LOQ: -	[120]

**Table 5:** Notifications from the RASFF Window database regarding exceedances of PA content in food products on the EU market (accessed on 31 May 2024).

No.	Product	Country of Origin	Notifying Country	Determined Level of PAs (µg/kg—ppb)	Maximum Level (µg/kg—ppb)	Notification Date
1	Psyllium Fibre Food Supplement	UK	Ireland	1177.0 ± 111.7 1113.5 ± 109.3	400	31 May 2024
2	Cumin	Turkey	Germany	8374 ± 3685	400	22 May 2024
3	Dill tops rubbed	Poland	Germany	1300 2000	400	16 May 2024
4	Ground oregano	Romania, Turkey	France	2563 ± 560	1000	7 May 2024
5	Dill	Poland, Spain	The Netherlands	840	400	3 May 2024
6	Cumin powder	Turkey	Bulgaria	3248.5 ± 1299.4 3232.5 ± 1293	400	29 April 2024
7	Herbes de Provence	France	France	2800 ± 700	600	19 April 2024
8	Ground cumin	Belgium	Belgium	773	400	18 April 2024
9	Pollen	France	Switzerland	3300	500	18 April 2024
10	Black tea—naturally flavoured maple	India	Belgium	347	150	15 April 2024
11	Dried oregano	Turkey	France	7861 ± 3931	1000	11 April 2024
12	Cumin seeds	Turkey	France	34,149.4 ± 17,074.7	400	11 April 2024
13	Cumin powder	Germany	Belgium	8860	400	29 March 2024
14	Oregano	Turkey	Switzerland	8062	1000	28 March 2024
15	Oregano	Turkey	Switzerland	24,231	1000	26 March 2024
16	Cumin	India	Czech Republic	985	400	11 March 2024
17	Black Tea	Kenya	Poland	540 ± 291	150	5 March 2024
18	Dried oregano	Belgium	France	1781.5	1000	5 March 2024
19	Gokshura/Lifepower/Karela	The Netherlands	The Netherlands	3920	400	28 February 2024
20	Kmin rzymski mielony (Ground cumin)	Poland	Poland	3340 ± 1169	400	22 February 2024
21	Herbata Czarna Earl Grey (Earl Gray Tea black tea)	Kenya	Poland	525 ± 180, 540 ± 291	150	20 February 2024
22	Cumin	India	Poland	1914 ± 670	400	20 February 2024
23	Parsley leaves	Poland	Romania	1400	400	15 February 2024
24	Kmin rzymski (Cumin)	Austria	Poland	776 ± 273	400	14 February 2024
25	Dried parsley leaves	Poland	Poland	3249 ± 459	400	26 January 2024
26	Oregano	Turkey	The Netherlands	2400	1000	19 January 2024
27	Green tea	Germany	The Netherlands	165	150	17 January 2024
28	Food Supplement	Norway	Sweden	1100	400	11 January 2024
29	Chamomile herbal tea	Czech Republic	Czech Republic	1936	400	10 January 2024
30	Pollen	Spain	Belgium	1430	500	9 January 2024
31	Oregano	Greece	The Netherlands	2600 ± 1300	1000	3 January 2024
32	Oregano	Turkey	The Netherlands	21,000	1000	28 December 2023
33	Ground cumin	Belgium	Belgium	752	400	22 December 2023
34	Oregano	Turkey	The Netherlands	1245	1000	21 December 2023
35	Oregano	Turkey	Poland	7941 ± 1571	1000	21 December 2023
36	Oregano	Jordan	Ireland	49,432.8 ± 5776.1	1000	21 December 2023
37	Cumin	Turkey	Germany	711	400	20 December 2023
38	Cumin, ground	Turkey	Germany	6080	400	13 December 2023
39	Black cumin seeds	Turkey	France	1054.6 ± 527.3	400	12 December 2023
40	Blackberry leaves	Albania	Germany	5170 ± 1293	200	8 December 2023
41	Mint tea (Mentha bruh, *Mentha piperita*)	Serbia	Croatia	>8550.5	400	5 December 2023
42	Dried oregano	Turkey	France	3626.4 ± 1813.2	1000	27 November 2023
43	Ground cumin	Spain	Belgium	2790	400	24 November 2023
44	Ground oregano	Greece	Germany	23,350	1000	24 November 2023
45	Chili powder	India, The Netherlands, Spain, Turkey	Belgium	2790	0	20 November 2023
46	Pollen	Spain	Belgium	1070	500	13 November 2023
47	Spiskummin (Cumin)	Lebanon	Sweden	1060, 1850, 2160	400	10 November 2023
48	Dried oregano	Turkey	Italy	3910 ± 773	1000	9 November 2023
49	Whole lovage leaf	Germany	The Netherlands	1310	1000	6 November 2023
50	Chives, grinded	Germany	The Netherlands	553	0	2 November 2023
51	Rosemary	France	The Netherlands	967	400	30 October 2023
52	Cumin and Organic Cumin	Egypt, India	Denmark	16,000, 1600	400	27 October 2023
53	Dried oregano	Turkey	Poland	3640 ± 1274	1000	26 October 2023
54	Cumin	Lebanon	Denmark	12,000	400	24 October 2023
55	Herbal tea	Morocco	Germany	594 ± 148	200	19 October 2023
56	Cumin seed	Turkey	Belgium	1306	400	9 October 2023
57	Cumin powder	Turkey	Bulgaria	>16,221	400	6 October 2023
58	Dried oregano	Turkey	Bulgaria	8640.7	1000	21 September 2023
59	Peppermint herbal tea	Poland	Czech Republic	657	400	20 September 2023
60	Oregano	Turkey	Luxembourg	3292 ± 745	1000	14 September 2023
61	Herbal infusion	China	Belgium	786	200	21 August 2023
62	Tarragon	France	Belgium	1120	400	11 August 2023
63	Cumin seeds crushed or ground	Turkey	Greece	2074 ± 415	400	7 August 2023
64	Dried oregano	Turkey	Greece	4285 ± 857	1000	7 August 2023
65	Kmin rzymski mielony (Cumin)	India, Poland	Poland	1217	400	11 July 2023
66	Ground cumin	Turkey	Belgium	23,813	400	26 June 2023
67	Ground cumin	Turkey	Greece	8281	400	26 June 2023
68	Cumin	Turkey	Germany	13,600	400	21 June 2023
69	Cumin	Turkey	Belgium	2259 ± 890	400	14 June 2023
70	Cumin seeds	Spain	Luxembourg	717 ± 108	400	14 June 2023
71	Herbata Loyd Earl grey	Poland	Poland	240 ± 40	150	12 May 2023
72	Ground cumin	Turkey	Bulgaria	1553.4	400	2 May 2023
73	Dried oregano	Turkey	Sweden	2263	1000	12 April 2023
74	Organic oregano, rubbed	Germany, Greece	Germany	24,000	1000	28 March 2023
75	Dried oregano	Poland	Czech Republic	1448	1000	28 March 2023
76	Oregano rubbed	Greece	Germany	17,000	1000	22 March 2023
77	Cumin grain	Belgium, France	France	10,000	400	7 March 2023
78	Cumin Whole	India	Ireland	527.1 ± 87.9	400	17 February 2023
79	Borage	Italy	Germany	>59,999	1000	7 February 2023
80	Ground cumin	Belgium, Syria	Belgium	16,596, 13,551.4	400	3 February 2023
81	*Ginkgo biloba* extract	France	Belgium	702	400	30 January 2023
82	Camomille tea	France	Belgium	2470	400	27 January 2023
83	Cumin seeds	Turkey	France	1148.9 ± 574.4, 660.9 ± 330.5, 563.7 ± 281.9	400	27 January 2023
84	Licorice root ground Zoethoutwortel gemalen	France	The Netherlands	1558	400	23 January 2023
85	Herbal tea mix	Morocco	Norway	11,608.3	200	12 January 2023
86	Black Tea (ceai negru)	Poland	Romania	700	150	10 January 2023
87	Pollen	Poland	Poland	1187 ± 301	500	3 January 2023
88	Cumin fines	Turkey	Spain	7290 ± 3650	400	29 December 2022
89	Ground cumin	-	Belgium	5298, 2926	400	16 December 2022
90	Dried oregano	Turkey	Poland	13,921 ± 2735	1000	13 December 2022
91	Cumin	-	Greece	17,512	400	13 December 2022
92	Ground cumin	India	Germany	4040 ± 1620	400	1 December 2022
93	Ground cumin	Afghanistan, France	Belgium	23,899, 14,249	400	22 November 2022
94	Oregano (dried)	Turkey	Belgium	1983.5	1000	21 November 2022
95	Dried oregano	Turkey	France	5174 ± 2587	1000	17 November 2022
96	Dried oregano	Turkey	Poland	8236 ± 1564	1000	15 November 2022
97	Ground cumin	-	Belgium	3697 ± 1395, 10,118 ± 3915	400	3 November 2022
98	Oregano	Greece	The Netherlands	30,313	1000	2 November 2022
99	Origano secco	Turkey	Italy	5591 ± 1177	1000	19 October 2022
100	Comino	Turkey	Spain	8170 ± 4090	400	11 October 2022
101	Ground cumin	Turkey	Switzerland	4436	400	10 October 2022
102	Dried oregano	Turkey	Bulgaria	>2500	400	10 October 2022
103	Ground cumin	Turkey	Ireland	1191.4 ± 197.8	400	25 August 2022
104	Cumin seeds	India	Switzerland	154,000, 2780, 14,100	400	15 June 2022
105	Cumin	Turkey	Sweden	12,350, 10,560	0	10 June 2022
106	Ground cumin	Turkey	Bulgaria	>2500	0	12 May 2022
107	Dried oregano	Turkey	Bulgaria	2154	400	10 May 2022
108	Dried oregano	Turkey	Bulgaria	2644.1	400	7 May 2022
109	Ground cumin	Turkey	Bulgaria	1505.4	400	24 April 2022
110	Ground Cumin	Turkey	Ireland	1723.8, 4810.6 ± 801.4	400	22 April 2022
111	Dried oregano	Turkey	Finland	6970	0	30 March 2022
112	Semillas de comino (Cumin seeds)	Turkey	Spain	50,000	400	7 March 2022
113	Ground cumin	Turkey	Czech Republic	11,907.7	0	1 March 2022
114	Organic bee feed	Spain	The Netherlands	97, 42, 880	500	5 January 2022
115	Oregano	Spain	Denmark	14,000 ± 5000	0	23 December 2021
116	Chamomile Tea	Uzbekistan	Denmark	5400	0	22 December 2021
117	Oregano	Turkey	Germany	2785, 2568	0	28 October 2021
118	Cumin seeds	Turkey	Germany	9474	0	19 October 2021
119	Oregano	Turkey	Switzerland	4879	0	2 June 2021
120	Oregano	Turkey	Germany	2079	0	20 May 2021
121	Organic cumin	Turkey	Germany	10,483.39	5000	14 May 2021
122	Cumin	Turkey	Germany	10,906.77	0	7 May 2021
123	Cumin	Turkey	Germany	10,406.94	5000	5 May 2021
124	Kräutertee (Herbal tea)	Czech Republic	Germany	2928.10	0	1 April 2021
125	Oregano	Turkey	Switzerland	8895	0	26 March 2021
126	Kreuzkümmel, gemahlen (Ground cummin)	Turkey	Germany	27,500 ± 970	0	12 February 2021
127	Kreuzkümmel, gemahlen (Ground cummin)	The Netherlands	Germany	21,200 ± 5300	0	21 January 2021
128	Ground cumin	Turkey	Switzerland	9948	0	24 December 2020
129	Ground cumin	Turkey	Switzerland	20,377, 5786	0	23 December 2020
130	Ground cumin	Turkey	Switzerland	5522	0	23 December 2020
131	Kreuzkümmel, gemahlen (Ground cummin)	Turkey	Germany	11,700 ± 2900	0	4 December 2020
132	Ground cumin	The Netherlands	Germany	55,176	0	18 November 2020
133	Cumin (Kreuzkümmel)	Lebanon	Germany	22,000, 18,900	0	18 November 2020
134	Anissamen (Anise seeds)	Egypt	Germany	12,184, 15,114, 1206 ± 188	0	20 August 2020
135	Cumin ganz	Syria	Germany	57,827	0	19 August 2020
136	Cumin, Organic	Turkey	Switzerland	29,120	0	30 June 2020
137	Bio Cumin	Turkey	Germany	56.100	0	30 April 2020
138	Ground cumin and dry oregano	Turkey	Denmark	15,000, 7200	0	24 April 2020
139	Oregano	Turkey	Germany	6620	0	30 March 2020
140	Dried camomile tea	Poland	Belgium	530	0	11 February 2020
141	Oregano getrocknet	Turkey	Germany	16,962 ± 8481	0	5 February 2020
142	Rubbed oregano	Turkey	Germany	8836	0	4 February 2020

## Data Availability

Not applicable.
